# On the design of a DEA-based device to pot entially assist lower leg disorders: an analytical and FEM investigation accounting for nonlinearities of the leg and device deformations

**DOI:** 10.1186/s12938-015-0088-3

**Published:** 2015-11-05

**Authors:** Shahram Pourazadi, Sadegh Ahmadi, Carlo Menon

**Affiliations:** MENRVA Group, School of Engineering Science, Faculty of Applied Science, Simon Fraser University, 8888 University Drive, Burnaby, BC V5A 1S6 Canada

**Keywords:** Dielectric elastomer actuator, Active compression bandage, Lower leg disorders, Calf compliance, Finite element modeling

## Abstract

**Background:**

One of the recommended treatments for disorders associated with the lower extremity venous insufficiency is the application of external mechanical compression. Compression stockings and elastic bandages are widely used for the purpose of compression therapy and are usually designed to exert a specified value or range of compression on the leg. However, the leg deforms under external compression, which can lead to undesirable variations in the amount of compression applied by the compression bandages. In this paper, the use of an active compression bandage (ACB), whose compression can be regulated through an electrical signal, is investigated. The ACB is based on the use of dielectric elastomer actuators. This paper specifically investigates, via both analytical and non-linear numerical simulations, the potential pressure the ACB can apply when the compliancy of the human leg is taken into account. The work underpins the need to account for the compressibility of the leg when designing compression garments for lower extremity venous insufficiency.

**Methods:**

A mathematical model is used to simulate the volumetric change of a calf when compressed. Suitable parameters for this calf model are selected from the literature where the calf, from ankle to knee, is divided into six different regions. An analytical electromechanical model of the ACB, which considers its compliancy as a function of its pre-stretch and electricity applied, is used to predict the ACB’s behavior. Based on these calf and ACB analytical models, a simulation is performed to investigate the interaction between the ACB and the human calf with and without an electrical stimulus applied to the ACB. This simulation is validated by non-linear analysis performed using a software based on the finite element method (FEM). In all simulations, the ACB’s elastomer is stretched to a value in the range between 140 and 220 % of its initial length.

**Results:**

Using data from the literature, the human calf model, which is examined in this work, has different compliancy in its different regions. For example, when a 28.5 mmHg (3.8 kPa) of external compression is applied to the entire calf, the ankle shows a 3.7 % of volume change whereas the knee region undergoes a 2.7 % of volume change. The paper presents the actual pressure in the different regions of the calf for different values of the ACB’s stretch ratio when it is either electrically activated or not activated, and when compliancy of the leg is either considered or not considered. For example, results of the performed simulation show that about 10 % variation in compression in the ankle region is expected when the ACB initially applies 6 kPa and the compressibility of the calf is first considered and then not considered. Such a variation reduces to 5 % when the initial pressure applied by the ACB reduced by half.

**Conclusions:**

Comparison with non-linear FEM simulations show that the analytical models used in this work can closely estimate interaction between an active compression bandage and a human calf. In addition, compliancy of the leg should not be neglected when either designing a compression band or predicting the compressive force it can exert. The methodology proposed in this work can be extended to other types of elastic compression bandages and garments for biomedical applications.

**Electronic supplementary material:**

The online version of this article (doi:10.1186/s12938-015-0088-3) contains supplementary material, which is available to authorized users.

## Background

Venous dysfunction in human lower extremities causes disorders that impact the life quality of considerable amount of individuals. Disorders such as orthostatic hypotension, edema and deep vein thrombosis are closely tied to the venous system failure to pump the blood back to the heart. Typically certain body mechanisms including vein valve and muscle contraction help the blood return. Sometimes these mechanisms fail to operate properly and lead to decrease in blood flow and an increase in blood volume accumulated in the lower extremities. In particular, any malfunction in capillary filtration causes abnormal balance between blood in capillaries and fluids in interstitial tissue. While standing, venous pressure in the lower leg increases which in turn results in an increase of oncotic pressure. This results in greater pressure gradient in the capillaries and thus more filtration. An increase in filtration causes accumulation of more fluid in interstitial tissue which results in edema [1]. Also, due to a higher compliance of venous, veins inflate while standing and result in venous pooling. Venous pooling can cause orthostatic intolerance and blood clots. It can also lead to chronic varicose veins, in which the valve mechanism in veins does not operate properly and blood tends to accumulate upstream.

In order to prevent and cure these disorders, different methods have been suggested [2]. The common way to treat venous pooling is to apply a compression to the lower extremities [3]. Compression to the lower part of the leg mimics the natural muscle contraction and helps blood return. It also increases the pressure of interstitial fluid and decreases the superficial venous pressure. Thus, the leakage of fluids to interstitial space is reduced [4]. In this regard, *compression stocking* (*CS*) is one of the recommended devices that apply mechanical compressions to the lower leg area. CS reduces venous hypertension and helps the leg muscle pump, which improves filtration, prevents skin breakdown, and eventually results in reduction of edema and venous pooling [5, 6]. Another mechanical device that prevents these disorders is *intermittent pneumatic compression* (*IPC*). IPC increases blood flow and improves blood return by simulating normal leg muscle activity [7]. The efficacy of CSs has been questioned [8, 9] and IPCs are typically bulky and therefore not suitable for ambulatory use. *Dielectric elastomer actuators* (*DEAs*) are introduced as *active compression bandages* (*ACBs*) to address the shortcomings of the previous devices [10]. In particular, DEAs can apply a variable (time-dependent) mechanical compression using an input electrical stimulus. Variable compression was shown to be more efficient than a static compression in different studies [11, 12]. Moreover, DEAs are lightweight, portable and very fast with experimental time responses as short as 2 ms [13].

DEAs are a category of electro-active polymers (EAPs) which have drawn much attention due to their simplicity, stability, fast response, high stretch ratio, high applicable force, and low cost [14]. Numerous studies are presented in literature that discuss the DEA applications and characterize them in various shapes and configuration such as roll [14], planar [15], cylindrical [16] and spherical [17, 18] actuators. A simple DEA unit consists of a dielectric elastomer that is sandwiched between a pair of compliant electrodes. Once voltage bias is applied to the DEA electrodes, the dielectric elastomer expands as a result of electrostatic Maxwell pressure across the dielectric. Considerable actuation strains and energy densities are achieved with DEAs which makes them interesting to be used for development of new emerging technologies [14].

The quantity of external compression is an important factor in treating the previously mentioned disorders on the lower extremities. For instance, it was shown that an external compression of 35–40 mmHg was required at the ankle to prevent edema in patients with severe venous disease [19]. Also [20] suggested that in order to optimize the venous flow in the lower leg, a graded compression of 18 mmHg at the ankle, 14 mmHg at mid-calf, and 8 mmHg at the knee is required. Thus, the bandages used for compression therapy should be designed to apply the proper range of compression. Typically, the amount of pressure exerted by elastic bandages is a function of the calf geometry. In practice, the calf is compressible and its volume and geometry change upon compression. Thus, the amount of compression that elastic bandages exert differs depending on the volume and shape of the calf. In this regard, Stenger et al. suggested that volumetric changes of the leg should be considered prior to the design of elastic bandages [3].Therefore, it is interesting to study and identify how profound the calf compressibility would affect the behavior of the DEA bandage.

In this paper, an analytical study and a numerical simulation based on the finite element method (FEM) are proposed to simulate the interaction between the ACB and the calf. The results of this study assist predicting how calf compressibility affects performance of an ACB and the use of this technology in future clinical studies.

## Mechanical properties of the calf

The human calf compresses upon application of pressure. The geometry and size of the calf change considerably with the application of an external compression. The relationship between changes in volume to changes in external pressure is often determined by its compliance (*C*):

1 $$ C = \frac{\delta \Delta V}{\delta P} $$

**Fig. 1:**
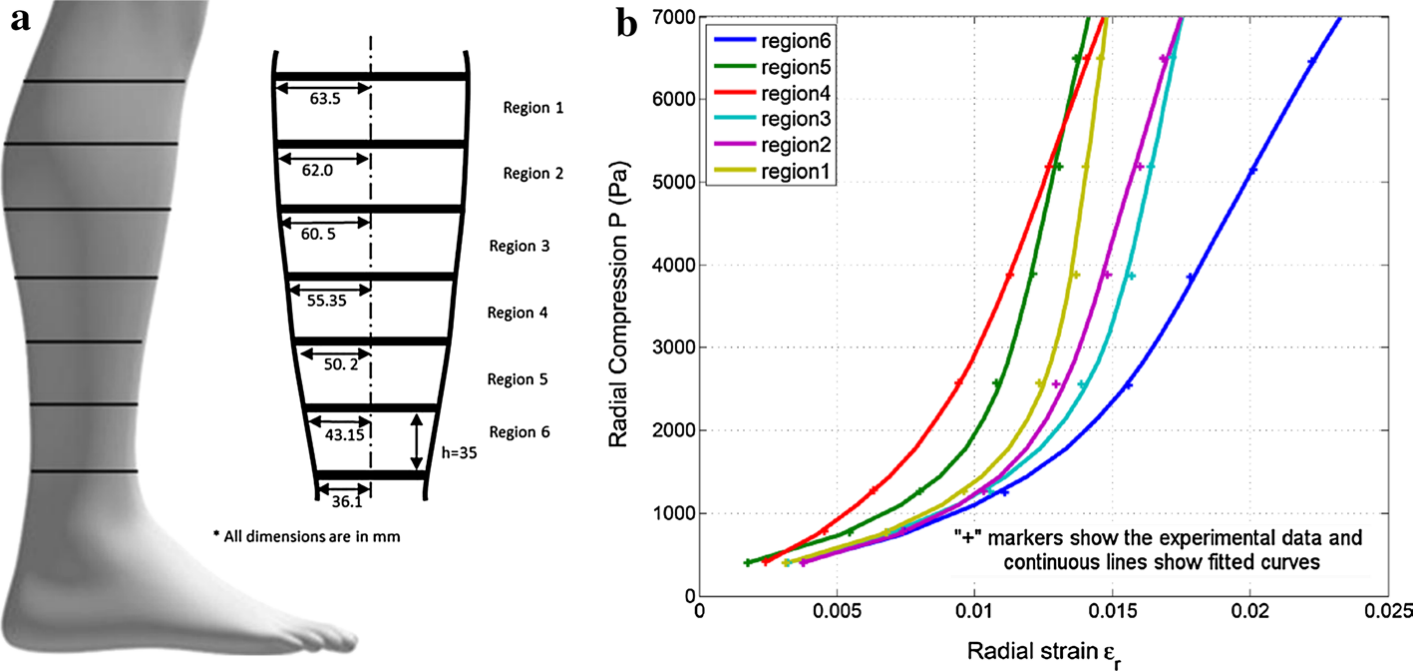
Human calf. **a** Conical geometry of the calf and its six different regions. Each region is assumed to be 35 mm apart in height [28].** b ** Fitted curves to calf compliance data obtained from [28] at the calf’s different regions

The compliance is a nonlinear function of external pressure and different regions of the calf show different values. There are several works in the literature that studied and measured the calf compliance with different methodologies [21–28]. The common clinical method of calf compliance measurement is to apply external pressure proximal to the knee with congestion cuffs and then monitor the volume changes in the calf area using plethysmography [21–25]. In this method it is assumed that the volume change in the limb is equivalent to volume change of the underlying venous vessel. This assumption however, is argued to be not very accurate [28]. Moreover, this method is believed to not precisely predict the compliance of the calf as the external pressure is applied proximal to the knee and not to the calf itself. A more sophisticated method was however used to accurately measure the calf compliance by monitoring cross sectional changes of the calf under application of an external pressure to the calf area [27, 28]. Specifically Thirsk et al. [28] proposed a procedure that removed possible artifacts, such as involuntary muscle contractions, from the measurements to obtain accurate measurements. In that work, compliance was measured in six regions of the leg, from the ankle to the knee (labeled as region 6 to 1 from now onward as it is shown in Fig. 1a, in 3 human subjects. The calf compliance was presented in the plots of calf cross sectional area change versus external pressure change.

In this work, the compliance data acquired by Thirsk et al. [28] was digitalized from their published report. The values of percentage change in the cross section area of the calf were divided by 2 to obtain the radial strain. This operation is considered to be a valid conversion assuming a uniform geometrical shape of the calf cross section [29]. True stress–strain data points were calculated from the pressure-radius change data set for each of the six regions of the calf. A curve fitting was then used to fit a best function for the relation between the true radial stress (*P*) in “Pa” on the calf and the true radial strain (*ε*_*r*_). The stress–strain curves for all calf regions are depicted in Fig. 1b. For all regions the best fitted curves were found to be an exponential function of the form presented in Eq. (2). The curves in Fig. 1b are continuation of curves that yield 0 strain at 0 pressure, thus a single exponential term cannot accurately follow this behavior and a second term was added in Eq. (2) to address this issue. The corresponding constant parameters are provided in Table 1

2 $$ \varepsilon_{r} = a \cdot e^{b \cdot P} + c \cdot e^{d \cdot P} . $$

**Table 1:** Constant parameters of the calf compliance relation expressed in Eq. 1

Region	a	b	c	d
6	0.013392	7.898E−05	−0.01600	−0.001137
5	0.009989	4.967E−05	−0.01465	−0.001386
4	0.008678	7.532E−05	−0.00913	−0.000828
3	0.01373	3.492E−05	−0.01686	−0.001133
2	0.01188	5.511E−05	−0.01466	−0.001392
1	0.012223	2.731E−05	−0.01513	−0.001263

## Analytical modeling of the DEA on a simulated human calf

**Fig. 2:**
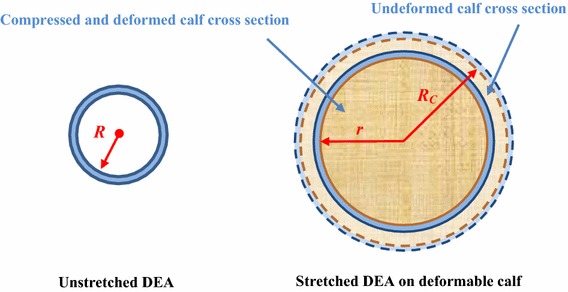
Changes of the ACB radius on compressible human calf

In this section, an analytical model of the cylindrical DEA [10, 16] is combined to the model of the human calf described by (2). The total pressure applied by the ACB has two components, the *mechanical pressure* which is the pressure resulting from the mechanical stress in the ACB before actuating the ACB, and the *actuation pressure,* which is the pressure variation after actuating the ACB. The ACB is assumed to be made out of a flat DEA with length *L* [11]. This flat DEA can be bent to form a cylinder, whose external radius is $R = \frac{L}{2\pi }$. The ACB, consisting of this DEA cylinder, is stretched radially as it is wrapped around the calf and the following stretch ratio *λ* can be used:

3 $$ \lambda = \frac{r}{R} $$

where *r* is the deformed radius of the ACB when it conforms the calf. Since the geometry of calf is conical, the ACB undergoes different stretch ratios along the height of the calf. In the analytical modeling of this work, it was assumed that the conical geometry of each calf region was consisted of small cylindrical geometries with finite height. The simulation procedure, which is explained in this section, is done for each of these small cylindrical geometries and the final values of radius and pressure was averaged over the entire calf region. As the stretch ratio of the ACB changes the amount of compression that is exerted on the calf is also changed. The amount of this compression is obtained using the following equation [16].

4 $$ P_{m} = \mathop \int \limits_{{\lambda_{b} }}^{{\lambda_{a} }} - \frac{{\mu_{1} \left( {\lambda^{{\alpha_{1} }} - \lambda^{{ - \alpha_{1} }} } \right) + \mu_{2} (\lambda^{{\alpha_{2} }} - \lambda^{{ - \alpha_{2} }} )}}{{\lambda (\lambda^{2} - 1)}}d\lambda $$

where *λ*_*a*_ and *λ*_*b*_ are stretch ratios of inner and outer radii of the cylindrical DEA respectively, and *μ*_1_, *μ*_2_, *α*_1_, *α*_2_ are the ogden parameters for the DEA material. As this compression from the ACB is applied, the calf radius changes. Assuming *R*_*c*_ as the radius of the undeformed calf (as shown in Fig. 2), λ_c_ is defined as the calf stretch ratio:

5 $$ \lambda_{c} = \frac{r}{{R_{c} }} = \frac{R}{{R_{c} }}\lambda $$

6 $$ \lambda_{c} = 1 - \varepsilon_{r} . $$

Substituting Eq. (2) in Eq. (6) and using Eq. (5), the calf radius after application of *P*_*m*_ can be obtained:

7 $$ r = R_{c} - R_{c} (ae^{{bP_{m} }} + ce^{{dP_{m} }} ). $$

In order to find the equilibrium state for *r* and *P*_*m*_, the set of coupled Eqs. (4) and (7) should be solved. However, this set of equations is nonlinear and neither *r* nor *P*_*m*_ can be written in terms of each other explicitly and a closed form analytical solution cannot be obtained. Hence, an iterative procedure is used to solve Eqs. (4) and (7) for the final calf radius *r* and external pressure *P*_*m*_ applied by ACB. In the first iteration of this procedure, the calf is undeformed and the ACB is stretched to *R*_*c*_. Using Eq. (4) a compression *P*_*m*_ is obtained. Then using Eq. (7) a new calf radius *r* is calculated. In the next iteration the *P*_*m*_ is again obtained using the new calf radius and consequently a newer calf radius is calculated. These iterations are continued until the value of *r* changes less than 0.01 % of the initial radius *R*_*c*_.

Once the bandage is wrapped and an equilibrium radius is reached for the calf and the ACB, the actuation phase is modeled and analyzed. The actuation pressure is calculated from the following equation [16]:

8 $$ P_{a} = \frac{{\varepsilon_{0} \varepsilon_{r} V^{2} }}{{2ln^{2} (b/a)}}\frac{{b^{2} - a^{2} }}{{a^{2} b^{2} }}. $$

As discussed earlier the total ACB pressure on the calf is composed of *P*_*a*_ and *P*_*m*_ and is given by:

9 $$ P_{t} = P_{m} - P_{a} $$

**Fig. 3:**
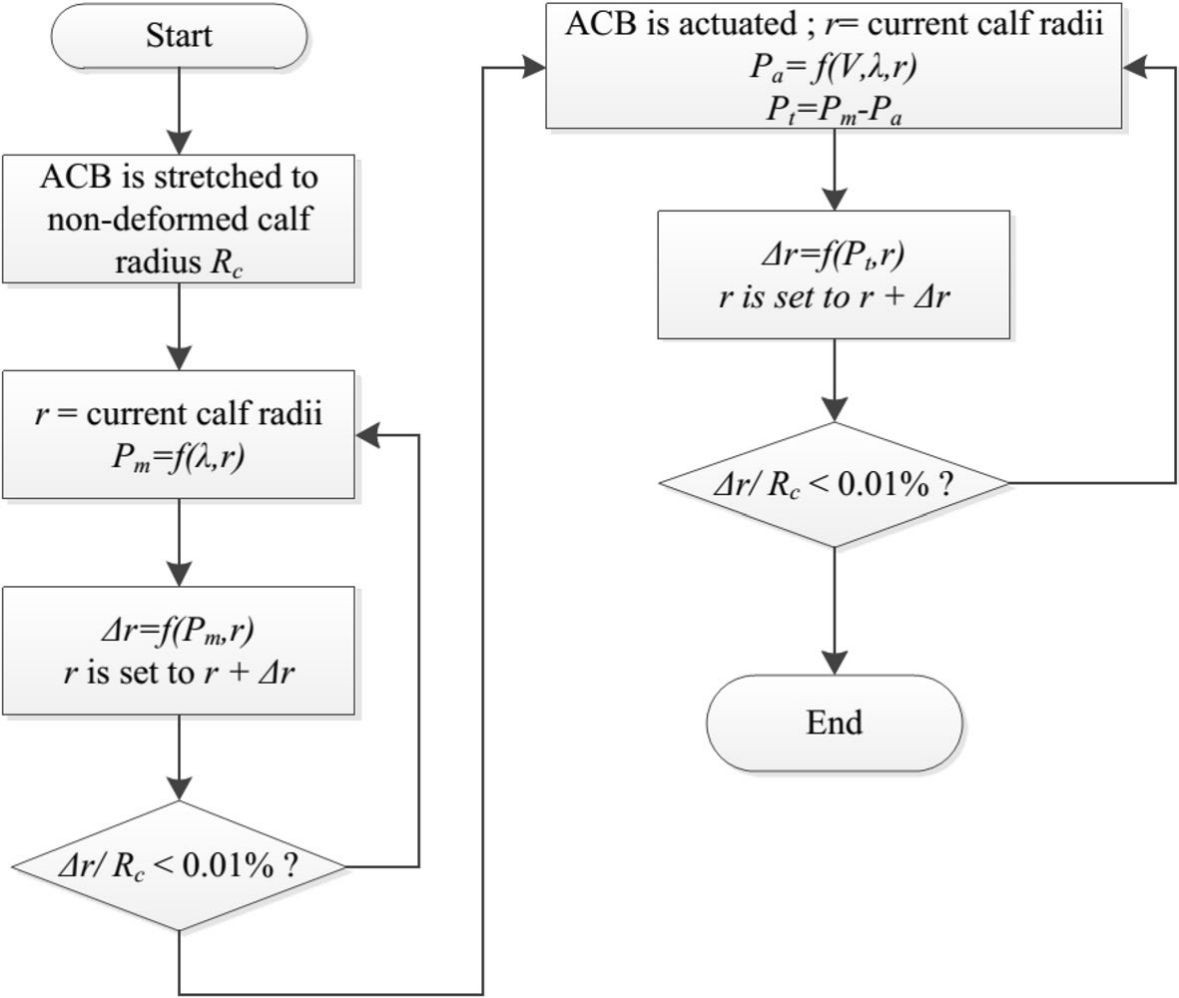
Flowchart of the analytical modeling of the ACB on the flexible calf

As it can be realized for Eq. (9), the ACB total pressure is reduced upon actuation. Thus, the radius of the calf is increased which in turn results in an increase of the ACB mechanical pressure. So we need to solve Eqs. (7) and (9) in order to find the final equilibrium state of the calf radius *r* and ACB total pressure *P*_*t*_. Again, this set of equation is solved similar to the previously mentioned iterative procedure. The steps of this iterative procedure are summarized in Fig. 3.

The developed analytical model gives us the ability to predict the behavior of the ACB on the flexible calf. Moreover, it provides us with a tool to design the ACB parameters such that desired compressions on the lower leg can be achievable. Simulations by the analytical model were carried out for the six regions of the calf and the results are discussed in the following sections.

## FEM analysis

Finite element modeling showed promising results for cylindrical and conical DEA geometries in the literature [10, 16]. Pourazadi et al. [10] used FEM to analyze the behavior of the ACB on a lower human leg geometry. In that work, the calf was assumed to be incompressible and the volume change effects in the lower leg region were neglected. However, the calf volume changes when external pressure is applied. To study the behavior of ACB on the compressible human calf, ANSYS software (Ansys Mechanical APDL 2011 *ANSYS Inc* V14.0) was used.

**Fig. 4:**
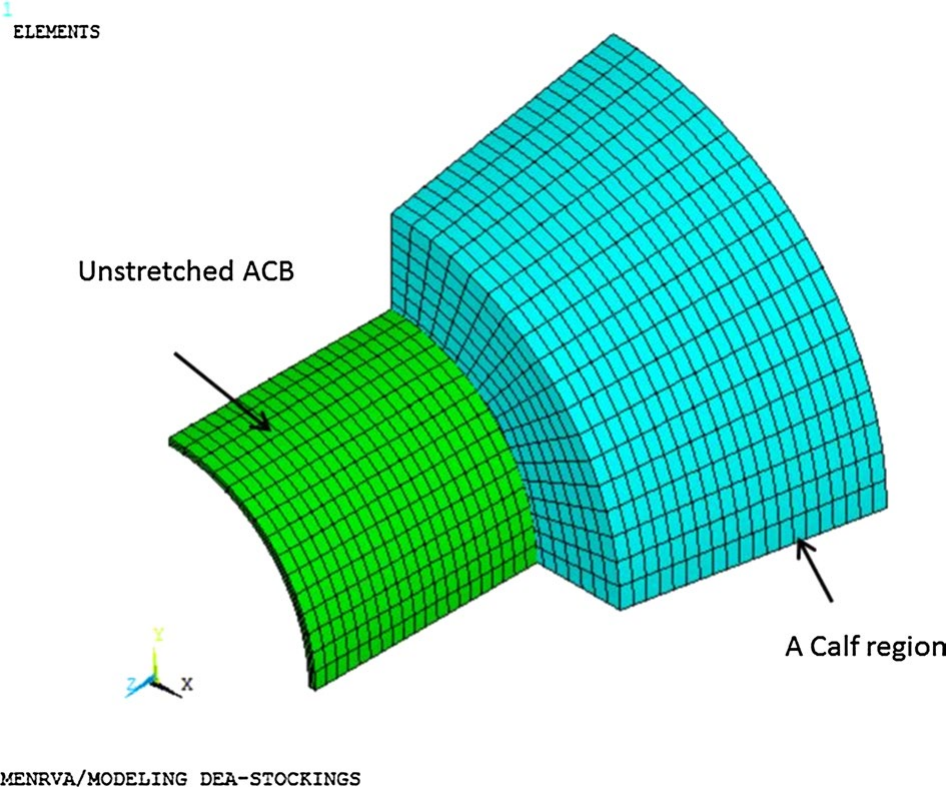
Geometry of the calf and ACB in FEM simulations

The ACB was originally modeled as a cylinder (as shown in Fig. 4) and a 2nd-order Ogden model was used to approximate its hyperelastic behavior. Ogden parameters from [10] were used. A truncated cone with specific dimensions (as shown in Fig. 4) was modeled for each calf region. A total of six regions were studied. The cone height for all regions was 35 mm. Every calf region had a different material property and required different nonlinear model parameters. A *Hyper*-*elastic Response Function* model was used to model the calf material in each region. In this model, the material’s compressibility is specified by *d*_1_ parameter—which is the inverse value of the bulk modulus. Further details of this material model, which is a built-in model in Ansys, can be found in Ansys documentation [30].

After creating the ACB and calf regions in the model, a fine FEM mesh was needed to be generated to obtain high-accuracy results. By selecting a finer mesh, although the accuracy of results was improved, the simulation time was rapidly increased as well. Therefore, an optimum mesh size was needed to be determined to achieve accurate results within a reasonable simulation time. As there was rotational symmetry for both the ACB and the calf geometries, only 1/4th of the geometries were modeled (as seen in Fig. 4). For the ACB, the geometry was meshed into 3 segments along the thickness, 15 segments along the circumference, and 20 segments along the height of the 1/4th cylinder. The calf geometry was also meshed into 18 × 18 × 20 segments respectively along its radial, circumferential and axial directions. Also, a total of 300 contact elements and 360 target elements were used in the model. To include the electro-active properties of the ACB in simulations, Solid226 (a higher order 3-D 20-node solid element with electrostatic material option) was selected. Also, Solid186 (a higher order 3-D 20-node solid element) was chosen for the calf geometry. For the contact areas of the ACB Conta174 (a 3-D 8-node surface-to-surface contact element) and for the calf’s contact area Targe170 (a 3-D target element) was used. Moreover, a frictionless contact was assumed between the solid geometries.

**Fig. 5:**
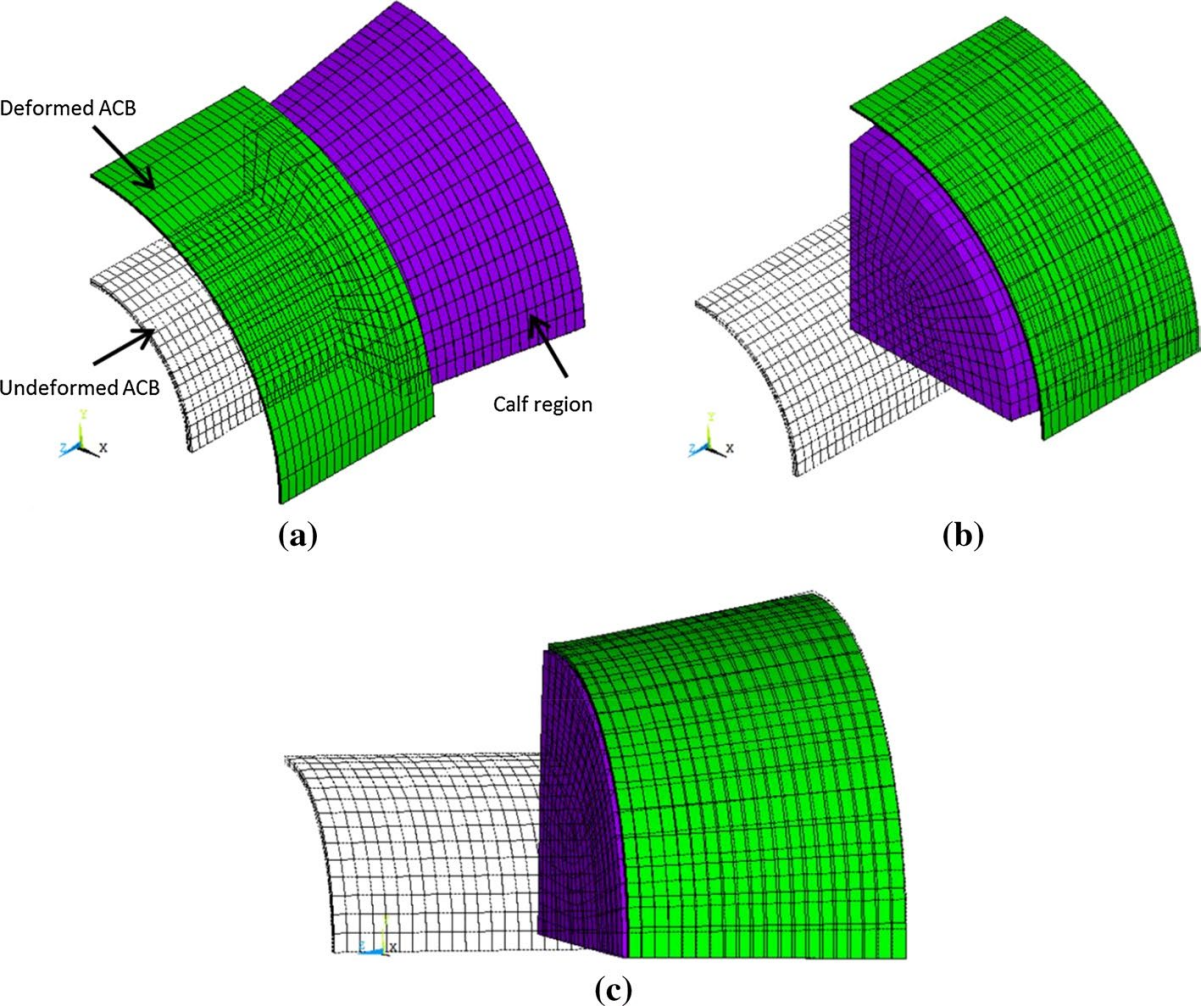
Deformation plots showing the position of the ACB and the calf region at the end of each FEM load step. **a** ACB is radially stretched to the maximum radius of the calf region; **b** ACB is positioned at the top of the calf region; **c** ACB is released to make contact with the calf region. Deformation plot after electrically actuating the ACB is similar to the plot **c** since the deformations after electrical actuation of the ACB is negligible. At each load step,* wireframe lines* show the geometries before deformation starts and* solid shapes* show the deformed geometries

In reality, an ACB bandage is wrapped around the calf and then is actuated. To avoid modeling a complicated contact problem and to reduce the calculation time, an alternative procedure which gave identical results was considered. The simulation steps were: (1) the sides of the ACB were fixed (to keep the height constant at 35 mm) and the ACB was stretched radially to the size of the largest radius of the calf region; (2) the stretched ACB was then displaced to perfectly locate on the calf region; (3) the radial constraint was removed to release the ACB and make contact with the calf region. The sides of both the ACB and calf segment were kept fixed and the contact pressure and volume changes were reported as outputs. The ratio of the average calf radius (mean of smaller and larger calf radii) to the initial ABC’s inner radius was defined as the stretch ratio (*λ*) which was chosen to vary from 1.4 to 2.2 in this study; (4) the ACB was actuated by applying an electrical potential difference of 11.3 kV between the top and bottom surfaces of the ACB. The pressure difference and volume changes were the outputs of this step. Simulation steps are shown in Fig. 5.

In the FEM setup explained above, there were 900 ACB elements, 6480 calf elements, and 660 contact elements. The total computation time for an ACB sample in contact with the calf region 6 varied from 5.5 to 9.5 h for stretch ratios of 1.4 and 2.2, respectively. It was found that the most time-consuming step is the 3rd step as the ACB makes initial contact with the calf region. A desktop PC with AMD FX™-8350 eight-core (4.7 GHz) processor and 16 GB of RAM was used. All CPU cores were fully utilized using ANSYS High Performance Computing (HPC) feature. To study the effect of mesh size on the accuracy of results, a second run was performed by selecting 4 × 20 × 25 = 2000 ACB elements, 20 × 20 × 25 = 10,000 calf elements, 500 contact elements, and 500 target elements. If the ACB sample was deformed to a stretch ratio of 2.2 the simulation finished after 42.5 h and the mechanical pressure after a full contact in step 3 was 6091.2 Pa. However, the coarser mesh size that was selected originally gave a nearly similar mechanical pressure, 6102.8 Pa. The relative error for such large pressures was less than 0.19 % with coarser mesh size but the calculation time was about 4.5 times shorter. Therefore, the coarser mesh size was chosen and simulations were performed for five different stretch ratios (range of 1.4–2.2 with 0.2 increments) and for all six calf regions. Results are discussed in the next section.

## Results and discussion

Different sizes of ACBs were investigated to study the effect of the initial length of the ACB and also cover a wide range of compressions on the calf. An ACB thickness of 1 mm with a dielectric constant of 7.41 was used for the simulations. Ogden parameters similar to [10] were used to simulate the hyperelastic behavior of the elastomer. Also, the ACB was actuated with 11.3 kV DC voltage for all simulations.

The initial lengths of the bandage were determined in order for the ACB to undergo a certain range of stretch ratio (i.e. 1.4–2.2) considering the non-deformed shape of the calf. However, as discussed earlier, the calf deforms under compression and thus the actual stretch ratio differs from the initial desired stretch ratio. In this work, the results are presented in terms of the initial desired stretch ratio for better consistency. Table 2 shows the values of the ACB lengths for the different calf regions and initial stretch ratios.

**Table 2:** Initial ACB lengths in centimetre

Calf region \Stretch ratio	1.4	1.6	1.8	2	2.2
1	28.2	24.6	21.9	19.7	17.9
2	27.5	24.1	21.4	19.2	17.5
3	26.0	22.7	20.2	18.2	16.5
4	23.7	20.7	18.4	16.6	15.1
5	20.9	18.3	16.3	14.7	13.3
6	17.8	15.6	13.8	12.4	11.3

**Fig. 6:**
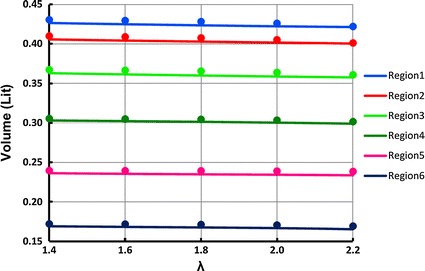
Analytical (*solid lines*) and FEM (*circle markers*) results for volume variations in various regions of the calf and different stretch ratios

The simulation results of the analytical and FEM modeling that were explained in “Analytical modeling of the DEA on a simulated human calf” and “FEM analysis” are followed in this section. Figure 6 shows the final volume of the different calf regions for different initial stretch ratios of the ACB after the ACB was wrapped and actuated. In this figure, the analytical and FEM results are represented with solid lines and circle markers, respectively. The maximum relative difference between the analytical and the FEM simulations is 2.4 % which is happening at region 6 for λ = 2.2. The detailed list of the volumes is listed in Additional file 1.

**Fig. 7:**
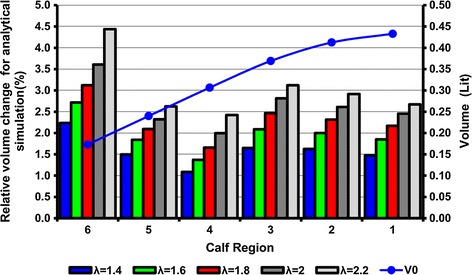
Relative change in the calf volume after wrapping and actuating the ACB. Each *color* represents a stretch ratio. The secondary axis on the *right hand side* shows the calf region initial volume indicated by the *solid line* with *circle markers*

**Fig. 8:**
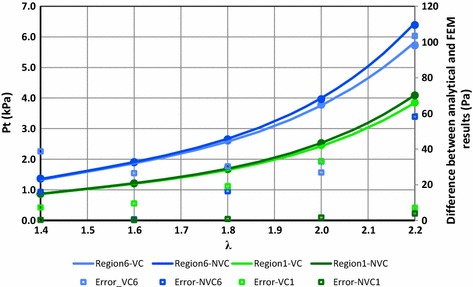
FEM (*circle markers*) and analytical (*solid lines*) results of the total pressure for regions 1 and 6

**Fig. 9:**
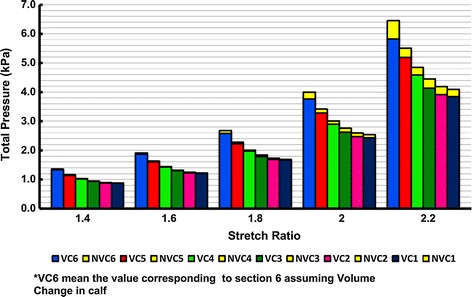
The ACB total pressure in all calf regions for different stretch ratios

**Fig. 10:**
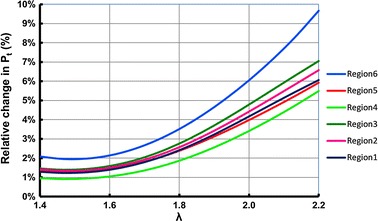
Percentage of change in the ACB total pressure between the two cases of VC and NVC

**Fig. 11:**
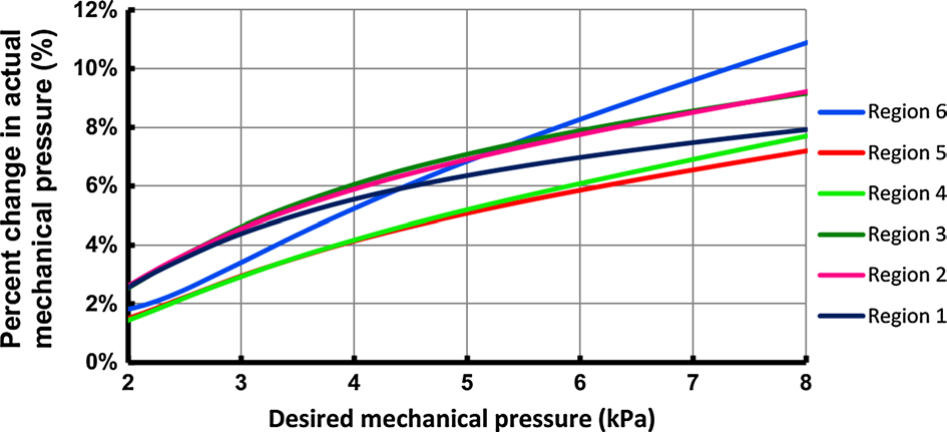
Difference between actual and expected values in mechanical pressure as a result of calf compressibility

**Fig. 12:**
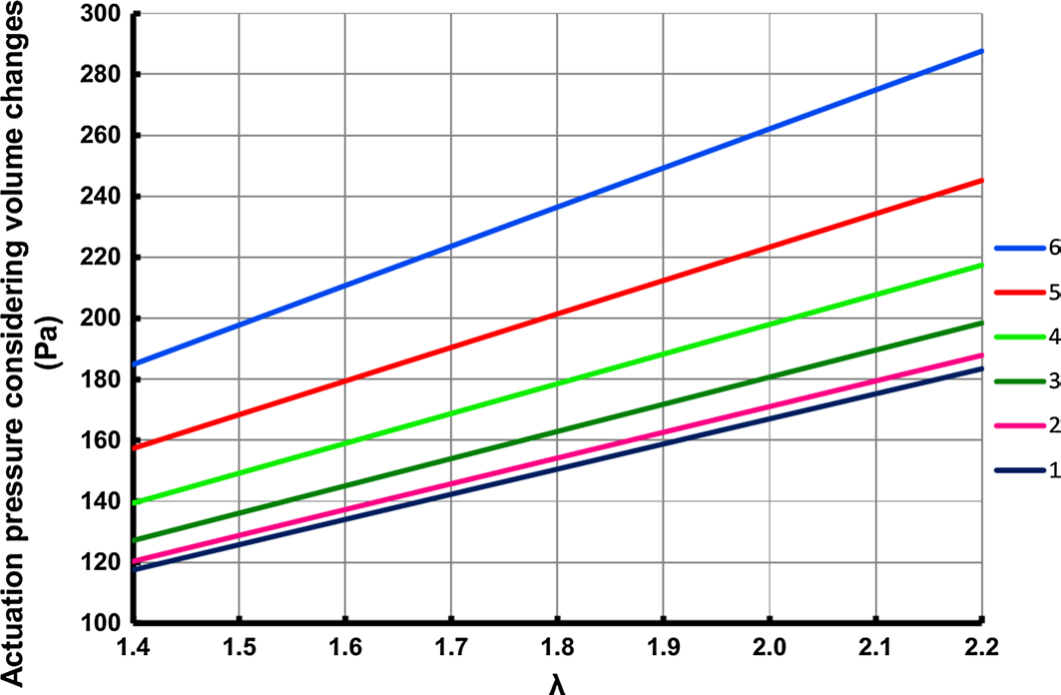
ACB actuation pressure for different calf regions

Figure 7 shows the relative change in the volume of the different calf regions (*E*_*v*_), defined by Eq. (10) versus different initial stretch ratios of the ACB.

10 $$ E_{v} = \frac{{V_{0} - V_{A} }}{{V_{0} }} \times 100 $$

where *V*_0_ is the initial calf volume and *V*_*A*_ is the calf volume after wrapping and actuation of the ACB that is given in Fig. 6. *V*_0_ is also plotted on the secondary axis of Fig. 7. The analytical simulations in Fig. 7 suggest that after wrapping and actuating the ACB the calf underwent a maximum of approximately 4.4 % volume change at the ankle region. For a certain stretch ratio, the maximum relative volume change took place in region 6 while the minimum was in region 4. The effect of volume changes on the performance of the DEA bandage is shown in Figs. 8, 9, 10, 11 and 12. Figure 8 shows the difference between the FEM and the analytical simulations for the total pressure on regions 1 and 6 for the two cases of considering a *volume change* (VC) and *no volume change* (NVC). The maximum difference between results from the analytical and FEM methods is 2.9 % at region 6 at λ = 1.4. The FEM simulation results shown in Figs. 6 and 8 verifies that the analytical model can closely estimate the ACB behavior on the human calf. A table of total pressure results is included in Additional file 2 for further reference.

Figure 9 shows the analytical results for the total pressure in all calf regions and illustrates the effect of considering volume change (VC) on the total pressure of the ACB. In this figure, each column group represents a specific initial stretch ratio for all sections of the calf. For each column, the bottom stack represents the total pressure after considering VC in the calf and the upper stack is the difference between considering VC and no volume change (NVC) in the calf. Thus, the combined height of each column represents the total pressure for the NVC case. Figure 9 shows that for a specific stretch ratio, the total pressure increases as we move from the knee to the ankle. It also shows that for a specific calf region the total pressure increases as the stretch ratio increases. However, the change in total pressure between the two cases of VC and NVC (shown by the second stack in each column/yellow color) is not uniformly decreasing or increasing and is a function of the compliance of the calf region. This change in total pressure follows the same pattern as the relative volume change in Fig. 7. Since region 4 has the lowest compliance (as shown in Fig. 1b), the change in total pressure is minimal for this region. These changes in the total pressure are also illustrated in Fig. 10. For a constant stretch ratio the change in total pressure is the lowest for region 4. The maximum change in total pressure corresponds to the ankle (region 6) and a 2.2 stretch ratio with 9.7 % change. This results implies that if the volume change in the ankle is considered, a total pressure of 5.82 kPa (43.6 mmHg) is achieved instead of 6.45 kPa (48.4 mmHg) which corresponds to a 630 Pa (4.7 mmHg) reduction in the total pressure. After region 6, region 3 has the highest change in total pressure with 7.1 % of change.

As mentioned earlier, the amount of pressure that the ACB exerts before electrical activation is denoted by mechanical pressure. The mechanical pressure of the ACB increases as the stretch ratio of the ACB increases. Also this mechanical pressure generally increases as the radius of the calf reduces [10]. Thus for a specific stretch ratio the amount of mechanical pressure is higher at the ankle section compared to other regions of the calf. One of the important rationales for this study is demonstrated in Fig. 11. Often the mechanical bandages are designed to provide a desired mechanical pressure. However, in practice, the amount of mechanical pressure changes due to the compressibility of the calf. In this case, the ACB can be stretched more to apply a higher amount of pressure and counteract the effect of volume change. Figure 11 shows the percent of change in actual mechanical pressure for a range of desired mechanical pressures. For example if the bandage is designed to provide a desired mechanical pressure of 4.90 kPa (36.8 mmHg) on the ankle, the actual mechanical pressure would be reduced by 6.7 % which is equivalent to 330 Pa (2.5 mmHg), making the actual mechanical pressure 4.57 kPa (34.3 mmHg). Variation of the actual compression from the desired compression by elastic bandages is an important fact that can lead to improper treatment of lower leg disorders [3]. Thus, it is proposed that the design mechanical pressure be increased by the percentage suggested in Fig. 11 to compensate for compression variations due to the changes in the calf volume.

In Fig. 12 the actuation pressure for the ACB, which is the pressure drop of the ACB once activated with 11.3 kV voltage, is shown for different sections of the calf. A voltage of 11.3 kV was used in order to conduct a study consistent with [10]. As it was also discussed in [10], the amount of activation pressure increased as the radius of the calf was reduced. As shown in Fig. 12, the actuation pressure changed almost linearly with respect to changes of stretch ratio. The maximum actuation pressure was 287.7 Pa (2.2 mmHg) which corresponded to the ankle region.

As mentioned earlier, applying a graded compression that reduces from ankle to knee is one of the recommended treatments for disorders associated with lower leg blood pooling [3]. One way to generate a compression that gradually reduces from ankle to knee is by making independent ACB short modules with different sizes for different calf sections. Each ACB module covers a single region of the calf and can be stretched independently, allowing for various compression profiles along the calf. Figure 9 provides the required stretch ratios that each ACB at each calf section should have in order to apply a desired compression profile. In this modular design, each single module could be actuated individually in order to generate an arbitrary variable compression such as peristaltic compression. This modular design, although challenging, provides a high degree of freedom to cover a wide range of dynamic compression profiles.

## Future improvements and limitations

This work investigated a recently proposed technology that potentially addresses some of the disadvantages of current compression therapy devices for the lower leg disorders. It should be noted that the investigated technology is still in its early stage of development, which justifies this study aimed at predicting the ACB behavior when a compressible human calf is considered.

The ACB faces some challenges and limitations that would need to be addressed in future studies. Specifically, DEAs are generally actuated with high actuation, which may pose limitations for use in wearable devices. The use of a stack of thin actuators could be used to address this challenge, as voltage could drastically be reduced. The use of a source not able to provide sufficient current to cause possible harm could also be considered.

Another aspect that should be considered is the prolonged use of ACB in contact with the human skin. Future studies should investigate the use of a layer of anti-allergic, breathable fabric laid at the interface between ACB and skin, which wicks away the skin sweat and moisture. Such fabrics are currently used in available compression garments [31]. The use of alternative materials for the ACB should also be studied.

Future studies should also consider the effect of the stress relaxation behavior of DEAs and its implications in the use of active compression garments, such as the one investigated in this work. It should in fact be noted that the stress relaxation in the ACB’s elastomer may potentially present a considerable reduction in the mechanical compression of the ACB over time. The amount of relaxation depends on the material of the elastomer and is a function of time, temperature and stress/strain level. Typically, the level of relaxation is very large and considerable in acrylic elastomers, however it is small particularly in silicone elastomers [14]. Typical acrylic elastomers show up to 500 % stress reduction after settling time of about half an hour for stretch ratios as low as 1.5 [32] while silicone elastomers can demonstrate less than 4 % stress reduction after settling time of 2 h for stretch ratios up to 2 [33]. Thus, the level of stress relaxation must be determined based on the material and the level of maximum stretch ratio used when wearing the ACB. Materials with low or negligible stress relaxation should be used in the fabrication of the ACB to lessen the reduction of mechanical compression on the limb due to the relaxation.

For the prototype investigated in this work, Fig. 10 shows that the ACB total pressure would reduce of around 4 % for a stretch ratio of 2 due to the sole calf compressibility. This amount of reduction in mechanical compression, as a result of the calf compressibility, would be comparable to the 4 % reduction in compression due to the stress relaxation [33]. Thus, it can be concluded that the effect of stress relaxation may considerably affect the performance of the ACB. While future investigations should address this aspect by investigating novel materials, simple pragmatic solutions could also be implemented. For instance, the ACB could be stretched more than needed when initially donned in order to compensate for the subsequent relaxation of the material. Alternatively, the ACB could be embedded with a suitable tightening mechanism, such as the Boa closure system [34]—this solution would enable the user to tighten the ACB when needed to compensate for the compression reduction due to the relaxation.

## Conclusion

In this paper the behavior of the active compression bandage (ACB) made out of dielectric elastomer actuator (DEA) was studied on a simulated human leg. ACBs could potentially be used in the treatment of different types of disorders associated with venous pooling in lower extremities such as orthostatic hypotension and edema. External compression on the lower leg helps balance the capillary filtration and venous return which in turn results in the reduction of venous pooling and normalizing the blood return. The literature suggests that a compression of 35–40 mmHg (4.67–5.33 kPa) at the ankle is required to prevent edema in patients with varicose veins. Also a graded compression of 18 mmHg (2.40 kPa) at the ankle, 14 mmHg (1.87 kPa) at mid-calf and 8 mmHg (1.07 kPa) at knee optimizes the blood flow in lower leg.

Since the human calf is flexible and compressible, the size and shape of the calf changes upon application of external compression. The literature was reviewed to obtain a relation between external pressure and change in volume of the calf (i.e. compliance). Different methods were discussed in this regard and finally a model for the relation between external compression and radial strain of the calf was developed based on [28]. This model was incorporated with analytical models available for the DEA. An iterative process was used to solve the system of equations. Also an FEM model was developed to simulate the contact problem between the ACB and the human calf. Results from the FEM model were used to cross validate the analytical simulations. The maximum relative difference between the FEM and the analytical modeling was as low as 2.9 %. The simulations demonstrated the values of the calf volume change under various compressions on different calf regions. For example, the calf underwent a 4.4 % volume change for an external pressure of 44 mmHg (5.82 kPa) at the ankle region. This change in volume can possibly be a considerable issue in analyzing stiff bandages with large young modulus.

In this paper it was shown that calf compressibility caused variations in the amount of compression exerted by elastic bandages. Specifically, the actual amount of compression by elastic bandages is different from the expected value. In the case of using the ACB, consideration of ankle flexibility resulted in approximately 9.7 % variations in the total pressure of the ACB for a case when the initial mechanical pressure of the ACB was 50 mmHg (6.70 kPa). However, results suggest that the effect of calf compressibility on the total pressure of the ACB was less than 5 % for initial mechanical pressures of less than 23 mmHg (3.07 kPa), 24.5 mmHg (3.27 kPa) and 27.5 mmHg (3.67 kPa) at the knee, mid-calf and ankle regions. Generally, the ACB with a specific value of stretch ratio, exerted higher mechanical pressure and resulted in higher actuation pressure for calf regions which had smaller sizes. The study in this work suggested that, the size and the compressibility of the calf should be taken into account in order to create a sufficiently accurate design of the elastic bandages that are supposed to exert a specific range of compressions.

## Additional files

10.1186/s12938-015-0088-3 Final calf volume values in liter after wrapping and actuating the ACB.
10.1186/s12938-015-0088-3 Total pressure values in kPa from the simulations for different stretch ratios and different calf regions.
